# Is methylphenidate truly cardiac-safe? Insight from biventricular speckle tracking echocardiography in pediatric patients with attention deficit hyperactivity disorder

**DOI:** 10.1007/s00431-026-07388-2

**Published:** 2026-09-18

**Authors:** Ahmed Osama Abd Rab El Rasool, Alshimaa Badreldeen, Doaa El Amrousy, Osama Younes

**Affiliations:** 1 Department of Neuropsychiatry, Faculty of Medicine, Tanta University, Tanta, Egypt; 2 Department of Pediatrics, Faculty of Medicine, Tanta University, Tanta, Egypt

**Keywords:** ADHD, Methylphenidate, Cardiac function, Children

## Abstract

Attention-deficit/hyperactivity disorder (ADHD) is a common neurodevelopmental condition identified by inattention, hyperactivity, and impulsivity. ADHD pharmacotherapy, particularly methylphenidate (MPH), has raised concerns about its cardiovascular safety. We aimed to assess the impact of methylphenidate on cardiac function in pediatric patients with ADHD using two-dimensional speckle tracking echocardiography (2D-STE). This study was performed on 40 children with newly diagnosed ADHD as the patient group and 40 age- and sex-matched healthy controls. Electrocardiographic screening and echocardiographic evaluations were conducted at baseline and after 6 months of MPH treatment. Heart rate (HR) and systolic and diastolic blood pressure (SBP, DBP) were also recorded. HR, SBP and DBP, were significantly elevated after treatment compared with their pre-treatment values. Diastolic functions of the left and right ventricles measured by tissue Doppler echocardiography (TDE) decreased significantly after treatment compared to their pretreatment values. Moreover, right and left ventricular systolic function measured by 2D-STE was significantly reduced after treatment compared to their pretreatment values. However, conventional echocardiography failed to detect any changes. None of our patients experienced any arrhythmias throughout the study duration.

*Conclusion*: Subclinical cardiac systolic and diastolic alterations were detected in pediatric ADHD patients treated with methylphenidate. The safety of the drug for the heart is warranted; therefore, careful patient selection, baseline cardiovascular assessment, and follow-up of these patients are strongly recommended.

**Table:** 

**What is Known:** • *Attention-deficit/hyperactivity disorder (ADHD) is a prevalent neurodevelopmental condition characterized by inattention, hyperactivity, and impulsivity.* • *ADHD pharmacotherapy, particularly methylphenidate (MPH), has raised concerns about its cardiovascular safety. No sufficient data about its cardiac safety in pediatrics.*	
**What is New:** • *We investigated, for the first time, the effect of methylphenidate on cardiac function in pediatric patients with ADHD using two-dimensional speckle tracking echocardiography.* • *We found subclinical cardiac systolic and diastolic alterations in pediatric ADHD patients treated with methylphenidate. The safety of the drug on the heart is warranted; therefore, careful patient selection, baseline cardiovascular assessment, and follow-up of these patients are strongly recommended.*

**What is Known:**

• *Attention-deficit/hyperactivity disorder (ADHD) is a prevalent neurodevelopmental condition characterized by inattention, hyperactivity, and impulsivity.*

• *ADHD pharmacotherapy, particularly methylphenidate (MPH), has raised concerns about its cardiovascular safety. No sufficient data about its cardiac safety in pediatrics.*

**What is New:**

• *We investigated, for the first time, the effect of methylphenidate on cardiac function in pediatric patients with ADHD using two-dimensional speckle tracking echocardiography.*

• *We found subclinical cardiac systolic and diastolic alterations in pediatric ADHD patients treated with methylphenidate. The safety of the drug on the heart is warranted; therefore, careful patient selection, baseline cardiovascular assessment, and follow-up of these patients are strongly recommended.*

## Introduction

Globally, attention-deficit/hyperactivity disorder (ADHD) represents a common neurodevelopmental disorder, affecting 5.9–7.1% of children and adolescents all over the world [1]. It is characterized by symptoms of inattention, hyperactivity, and executive dysfunction, which can significantly impair daily functioning [2]. The etiology of ADHD involves biochemical, psychological, genetic, and environmental components [3]. Growing evidence suggests that chronic inflammation may play a critical role in its pathophysiology [4].

While ADHD is primarily managed through behavioral interventions and pharmacotherapy, stimulant medications such as amphetamines and methylphenidate (MPH) remain the cornerstone of treatment [5]. These agents increase synaptic dopamine levels, thereby improving attention and reducing impulsivity [6]. However, concerns have arisen regarding their cardiovascular safety, as they are associated with elevated blood pressure (BP) and heart rate (HR) [7]. Although these effects are generally mild, the potential for serious adverse events, including arrhythmias, atherosclerosis, and myocardial ischemia in adulthood, remains controversial [8–10].

Increased visits to the emergency room (ER) due to cardiac symptoms were observed in children receiving MPH treatment [11]. Moreover, sudden cardiac arrest and cardiac dysfunction were reported in ADHD patients, especially children, receiving MPH [12–14]. Given the chronic nature of ADHD and its treatment, the impact of both the disorder and its pharmacotherapy on cardiac function is essential to be evaluated. However, studies in pediatrics are lacking. Hence, this study aimed to investigate the effects of MPH treatment on cardiac function in pediatric patients with ADHD.

## Methods

### Study design and setting

This cohort study was conducted at the Neuropsychiatry Department, the Psychiatry and Neurology Center, and the Cardiology Unit of the Pediatrics Department in one of the University Hospitals from March 2024 to December 2024.

### Participants

The study included 80 participants divided into two groups:

- Patient group: included 40 children and adolescents newly diagnosed with ADHD according to Diagnostic and Statistical Manual of Mental Disorders, Fifth Edition (DSM-5) criteria [1] who were planned to start treatment with osmotic-release oral system (OROS)-MPH at a starting dose of 18 mg/day once daily.
- Control group: included 40 age- and sex-matched healthy controls without any psychiatric or medical conditions. They were recruited from family members, outpatients, friends, or classmates of the study subjects with ADHD.

Inclusion criteria: pediatric patients newly diagnosed with ADHD aged between 7 and 18 years who were planned to start MPH treatment and could perform study-related tasks.

Exclusion criteria: ADHD patients already on treatment, a history of hypoxic-ischemic events or central nervous system infections, congenital or acquired heart disease, comorbid psychiatric disorders (e.g., autism spectrum disorder, schizophrenia), serious medical illnesses (e.g., epilepsy), congenital anomalies, endocrine diseases, obesity, or an IQ below 80.

### Procedures

All participants underwent comprehensive evaluations, including:

1. Thorough history taking, such as age, sex, and medical history.
2. Complete a general and systematic examination, especially a neurological examination, to exclude neurological and systemic illnesses.
3. Anthropometric measurements, including weight, height, and body mass index, were performed. Vital signs such as BP and HR were also measured before and 6 months after the treatment.
4. Neuropsychological assessment:
  Stanford–Binet Intelligence Test (5th edition): This test is used for the diagnosis of developmental or intellectual disabilities in young children. It consists of both verbal and nonverbal subtests and measures five weighted factors: knowledge, quantitative reasoning, visual-spatial processing, working memory, and fluid reasoning. Each of the five factors is assigned a weight, and the combined score is often reduced to a ratio commonly known as the intelligence quotient (IQ) [15, 16].
  Kiddie Schedule for Affective Disorders and Schizophrenia (K-SADS): It is a structured diagnostic interview used in clinical and research settings to assess psychiatric disorders in children and adolescents [17, 18].
  Conners’ Rating Scale for ADHD: It was given to the parents and teachers for rating the degree of severity of the children’s ADHD symptoms [19, 20].
  A socioeconomic status scale [21].
5. Cardiac evaluation:
  12-lead ECG monitoring: to detect any rhythm abnormalities.
  Transthoracic echocardiography was carried out using a widely available ultrasound system equipped with an appropriate transducer (Vivid 7 or Vivid 9, GE Healthcare, Horten, Norway) with 3.5-MHz, S7 and V3 matrix real-time two-dimensional probes. Acquired digital loops were stored on the echocardiographic system hard disk and subsequently transferred to a workstation (Echo PAC PC, 112 and 113; GE, Horten, Norway) for offline evaluation. Conventional echocardiography, tissue Doppler imaging (TDI), and two-dimensional speckle tracking echocardiography (2D-STE) were performed to assess cardiac function.

#### Conventional echocardiograph

Left ventricular ejection fraction (LV EF) was measured by M mode, where LV EF % = left ventricular end-diastolic volume (LV EDV)—left ventricular end-systolic volume (LV ESV)/LV EDV. Left and right ventricular diastolic function was assessed using LV E/A and RV E/A ratio (a ratio of early to late peak mitral and tricuspid flow velocities, respectively), while tricuspid annular plane systolic excursion (TAPSE) using the M mode in the apical 4-chamber view was measured to assess the right ventricular systolic function.

#### Tissue doppler imaging (TDI)

Left and right ventricular systolic function was assessed by measuring the peak systolic (*S*′) velocity. Diastolic function was evaluated using the ratio of early to late diastolic myocardial velocities (*e*′/*a*′). Furthermore, global ventricular performance was quantified using the myocardial performance index (MPI). The MPI was calculated according to the following equation: MPI = (ICT + IRT)/ET.

where ICT represents isovolumetric contraction time, IRT denotes isovolumetric relaxation time, and ET corresponds to ejection time.

#### Two-dimensional speckle tracking echocardiography (2D-STE)

2D-STE images were acquired at a frame rate exceeding 40 frames per cardiac cycle, over three consecutive cardiac cycles. Following adjustment of the two-dimensional views, automated endocardial border detection was performed using dedicated software. When necessary, manual correction of the tracking was applied to ensure optimal accuracy. Global longitudinal strain (GLS) of both LV and RV was subsequently quantified. Reproducibility was evaluated by assessing intra- and inter-observer variability in a subset of 20 randomly selected patients. This was achieved through repeated measurements conducted by the same observer and by a second experienced pediatric cardiologist, both of whom were blinded to group allocation.

ADHD patients were treated with an osmotic-release oral system (OROS)-MPH for 6 months. Cardiac evaluations were performed before and 6 months after treatment to monitor changes.

### Ethical considerations

The Ethics Committee of our Faculty of Medicine approved the study. Informed consent was obtained from the guardians of all included children.

### Statistical analysis

SPSS version 21 software (SPSS Inc., Chicago, IL, USA) was used to perform the statistical analysis. Descriptive data were expressed as numbers and percentages. While numerical data were expressed as mean and standard deviation (SD) if normally distributed. The Shapiro–Wilk test was used to test the normality of the data. The chi-square test was used to compare descriptive data between the two groups. Student’s *t*-test was used for the comparison of normally distributed numerical data between the two groups. A paired *t*-test was used to compare numerical data within the patient group before and after treatment. A *P*-value was considered significant if it was less than 0.05.

## Results

Table 1 shows that there were no statistically significant differences between the two groups as regards age, sex, BMI, residence, family type, or socioeconomic status. The absence of significant demographic discrepancies ensures that these factors did not confound the study outcomes. However, all psychometric measurements of Conners’ rating scale were significantly higher in the patient group compared to the control group (*P* = 0.001).

**Table 1:** Demographic and clinical data of the studied groups

Variables	ADHD Group	Control Group	*P*
Age (years)	9.5 ± 2.3	9.6 ± 2.4	0.859
Sex (male)	28 (70%)	26 (65%)	0.732
BMI (kg/m^2^)	17.2 ± 2.5	16.9 ± 2.1	0.146
Residence (urban)	16 (40%)	18 (45%)	0.713
Family type (nuclear)	28 (70%)	26 (65%)	0.654
Socioeconomic status (low)	26 (65%)	24 (60%)	0.634
Conners’ teacher rating score Conners’ parent rating score Inattention score Hyperactivity score Impulsivity score	72.3 ± 10.5 76.5 ± 12.1 19.2 ± 4.6 22.1 ± 5.3 18.4 ± 4.1	42.1 ± 7.8 40.5 ± 6.9 8.1 ± 2.4 7.6 ± 2.1 6.9 ± 1.8	0.001 0.001 0.001 0.001 0.001

*BMI*, body mass index

Table 2 presents the prevalence of psychiatric comorbidities identified using the K-SADS interview. ADHD children exhibited significantly higher rates of anxiety disorders, oppositional defiant disorder (ODD), conduct disorder, and learning disabilities compared to controls. These findings underscore the high prevalence of comorbid conditions in ADHD, emphasizing the need for comprehensive clinical assessments and multidisciplinary management approaches.

**Table 2:** K-SADS psychiatric comorbidities in the studied groups

Comorbidity	ADHD group (*n* = 40)	Control group (*n* = 40)	*P-*value
Anxiety disorder	14 (35%)	4 (10%)	0.03
Oppositional defiant disorder (ODD)	16 (40%)	0 (0%)	0.01
Conduct disorder	10 (25%)	0 (0%)	0.02
Learning disability	12 (30%)	2 (5%)	0.03

Both left and right ventricular systolic and diastolic functions were comparable in both groups before treatment, as assessed by different echocardiographic modalities. Moreover, SBP, DBP, and HR were insignificantly different between the patient and control groups at baseline **(**Table 3**)**.

**Table 3:** Cardiac function in the studied groups before treatment

Variables	ADHD group	Control group	P
Left ventricular function			
LV FS%	39.6 ± 2.1	40.4 ± 3.3	0.337
LV EF%	75.2 ± 6.8	76.3 ± 4.4	0.555
Mitral E/A ratio	1.7 ± 0.2	1.6 ± 0.3	0.229
LV S	7.1 ± 0.7	6.9 ± 0.7	0.504
Mitral *e*′/*a*′	1.8 ± 0.2	1.8 ± 0.2	0.236
LV MPI	0.39 ± 0.05	0.40 ± 0.04	0.787
2D-LV GLS	− 21.1 ± 1.8	− 21.3 ± 2.2	0.696
Right ventricular function			
TAPSE	21.5 ± 3.3	20.9 ± 2	0.525
RV S	9.5 ± 0.9	9.1 ± 1.1	0.236
RV *e*′/*a*′	1.5 ± 0.1	1.7 ± 0.4	0.087
RV MPI	0.39 ± 0.06	0.36 ± 0.06	0.114
RV 2D-GLS	− 22.8 ± 1.3	− 22.5 ± 1.6	0.452
Vital signs			
SBP (mmHg)	97.4 ± 11.6	98.3 ± 10.8	0.864
DBP (mmHg)	66.5 ± 8.5	65.9 ± 7.7	0.981
HR (b/m)	79.2 ± 5.8	80.0 ± 3.4	0.346

*LV FS*, left ventricular fraction shortening; *LV EF*, left ventricular ejection fraction; *E/A*, early/late diastolic velocities; *LV S*, left ventricular systolic wave; *e*′/*a*′, early/late diastolic velocities by tissue Doppler imaging; *MPI*, myocardial performance index; *2D LV-GLS*, two-dimensional left ventricular global longitudinal strain; *TAPSE*, tricuspid annular plane systolic excursion; *2D RV-GLS*, two-dimensional left ventricular global longitudinal strain; *SBP*, systolic blood pressure; *DBP*, diastolic blood pressure; *HR*, heart rate

Table 4 shows that Mitral *e*′/*a*′, LV MPI, 2D LV-GLS, RV *e*′/*a*′, RV MPI, and 2D RV-GLS significantly decreased in the patient group after 6 months of treatment compared to the control group. However, conventional echocardiographic parameters failed to show any significant changes. Interestingly, SBP, DBP, and HR were significantly higher in the patient group after treatment compared to the control group.

**Table 4:** Cardiac function and vital signs in the studied groups after treatment

Variables	ADHD group	Control group	*P*
Left ventricular function			
LV FS%	39.7 ± 5	40.5 ± 3.2	0.356
LV EF%	71.9 ± 5.8	75.3 ± 4.5	0.123
Mitral E/A ratio	1.6 ± 0.3	1.7 ± 0.3	0.080
LV S	7.0 ± 1.0	6.9 ± 0.8	0.504
Mitral *e*′/*a*′	1.6 ± 0.1	1.8 ± 0.3	0.005
LV MPI	0.47 ± 0.10	0.39 ± 0.04	0.02
2D-LV GLS	− 18.7 ± 2.9	− 21.9 ± 2.4	0.001
Right ventricular function			
TAPSE	20.6 ± 1.9	20.7 ± 2.2	0.172
RV S	9.1 ± 1.0	9.2 ± 1.3	0.298
RV *e*′/*a*′	1.2 ± 0.2	1.7 ± 0.5	0.001
RV MPI	0.45 ± 0.07	0.35 ± 0.04	0.001
RV 2D-GLS	− 18.9 ± 1.1	− 22.7 ± 1.4	0.001
Vital signs			
SBP (mmHg)	111.5 ± 10.5	96.7 ± 10.2	0.001
DBP (mmHg)	80.1 ± 4.3	68.7 ± 5.6	0.001
HR (b/m)	90.4 ± 6.1	79.9 ± 7.2	0.001

*LV FS*, left ventricular fraction shortening; *LV EF*, left ventricular ejection fraction; *E/A*, early/late diastolic velocities; *LV S*, left ventricular systolic wave; *e*′/*a*′, early/late diastolic velocities by tissue Doppler imaging; *MPI*, myocardial performance index; *2D LV-GLS*, two-dimensional left ventricular global longitudinal strain; *TAPSE*, tricuspid annular plane systolic excursion; *2D RV-GLS*, two-dimensional left ventricular global longitudinal strain

Table 5 shows that mitral *e*′/*a*′, LV MPI, and 2D-LV-GLS significantly decreased after treatment compared to their values before treatment in the patient group, indicating LV diastolic and systolic dysfunction. However, conventional echocardiographic parameters such as LV EF, LV FS, and mitral E/A ratio failed to show any significant changes. Moreover, RV MPI, RV *e*′/*a*′, and 2D-RV-GLS significantly decreased after treatment in the patient group, indicating right ventricular dysfunction.

**Table 5:** Cardiac function and vital signs in the patient group before and after treatment

Variables	Patient before treatment	Patient after treatment	*P*
Left ventricular function			
LV FS	39.6 ± 2.1	39.7 ± 5	0.912
LV EF	75.2 ± 6.8	71.9 ± 5.8	0.111
Mitral E/A ratio	1.7 ± 0.2	1.6 ± 0.3	0.069
LV S	7.1 ± 0.7	7.0 ± 1.0	0.512
Mitral *e*′/*a*′	1.8 ± 0.2	1.6 ± 0.1	0.005
LV MPI	0.39 ± 0.05	0.47 ± 0.10	0.002
2D LV- GLS	− 21.1 ± 1.8	− 18.7 ± 2.9	0.001
Right ventricular function			
TAPSE	21.5 ± 3.3	20.6 ± 1.9	0.636
RV S	9.5 ± 0.9	9.1 ± 1.0	0.254
RV *e*′/*a*′	1.5 ± 0.1	1.2 ± 0.2	0.01
RV MPI	0.39 ± 0.06	0.45 ± 0.07	0.002
RV 2D-GLS	− 22.8 ± 1.3	− 18.9 ± 1.1	0.001
Vital signs			
SBP (mmHg)	97.4 ± 11.6	111.5 ± 10.5	0.001
DBP (mmHg)	66.5 ± 8.5	80.1 ± 4.3	0.001
HR (b/m)	79.2 ± 5.8	90.4 ± 6.1	0.001

*LV FS*, left ventricular fraction shortening; *LV EF*, left ventricular ejection fraction; *E/A*, early/late diastolic velocities; *LV S*, left ventricular systolic wave; *e*′/*a*′, early/late diastolic velocities by tissue Doppler imaging; *MPI*, myocardial performance index; *2D LV-GLS*, two-dimensional left ventricular global longitudinal strain; *TAPSE*, tricuspid annular plane systolic excursion; *2D RV-GLS*, two-dimensional left ventricular global longitudinal strain; *SBP*, systolic blood pressure; *DBP*, diastolic blood pressure; *HR*, heart rate

Regarding vital signs, SBP, DBP, and HR significantly increased in the patient group after treatment compared to their pretreatment readings **(**Table 5**)**. No rhythm abnormalities were detected before or during the treatment period in the patient group.

## Discussion

Pharmacotherapy, especially MPH, is the first-line treatment in school-aged pediatric patients with ADHD. However, its safety on the cardiovascular system in pediatrics is still questionable, and pediatric studies on this topic are limited [22]. To the best of our knowledge, our study is the first to assess cardiac function in pediatric patients with ADHD who were on MPH treatment using 2D-STE. Our analysis revealed there is a subtle cardiac alteration in these patients.

Several studies reported a significant increase in HR and BP after treatment with MPH in pediatric patients with ADHD with short-term stimulant use, and with up to one year of use [23–26]. We found that HR, as well as SBP and DBP, were significantly elevated after 6 months of treatment compared to their pretreatment readings. Most of these elevations are within the normal limits, but the probability of an increased risk of hypertension in the future is still considerable.

CNS stimulants such as MPH exert their effects by inhibiting the presynaptic reuptake of norepinephrine and dopamine, while also promoting their release into the extraneuronal space. The resulting elevation in circulating catecholamines stimulates cardiovascular β1-adrenergic receptors, leading to an increase in heart rate. In parallel, activation of α-adrenergic receptors induces peripheral vasoconstriction, which subsequently contributes to elevated blood pressure [27–29].

After treatment, we found that left and right ventricular diastolic function decreased significantly as revealed by TDI. Methylphenidate has been associated with cases of pulmonary hypertension (PH), including the pediatric population [30]. PH, in turn, increases right ventricular afterload, leading to structural remodeling and impaired right ventricular diastolic function. Therefore, RV diastolic dysfunction observed in our cohort may be partially explained by subclinical or early pulmonary vascular changes induced by the drug. Similarly, increased systolic and diastolic blood pressure detected in our patients could be responsible for the LV diastolic dysfunction detected in our cohort.

Interestingly, the observed impairment in both left and right ventricular speckle tracking-derived strain in children receiving methylphenidate suggests the presence of subclinical myocardial alterations, even in the absence of overt clinical cardiac abnormalities. 2D-STE represents an advanced echocardiographic technique that enables the detection of early, subclinical myocardial dysfunction across a wide range of pediatric conditions before changes become evident with conventional echocardiographic parameters [31–33].

There are no previous similar studies in the literature to compare our results with, except for one study by Kara et al. [34], who evaluated the relationship between long-term use of MPH in pediatric patients with ADHD and cardiac function using TDI. They found that LV diastolic function was significantly lower than that of the control group.

Interestingly, a case report documented the occurrence of acute cardiomyopathy and pericarditis with LV EF of 30–35% and increased cardiac biomarkers in a 17-year-old adolescent after a single dose of MPH [35]. Similarly, an 18-year-old male diagnosed with ADHD and treated with MPH for one year presented to the ER with dyspnea and hypoxia, found to have severe dilated cardiomyopathy and biventricular failure with LV EF 20–25%, the LV was hugely dilated with moderate mitral regurgitation [36]. This raised concerns about the safety of MPH for the heart in these patients, irrespective of treatment duration.

Cardiac dysfunction in our patients may be explained by several mechanisms. One of these mechanisms is the long-term elevation of catecholamine levels mediated by MPH treatment, with a resultant increase in HR and BP. Excessive prolonged exposure to catecholamines can lead to overexpression of cardiac beta-1 and alpha-1 receptors with subsequent myocyte damage and necrosis, myocardial hypertrophy, increased LV filling pressure and oxygen consumption, and dilated cardiomyopathy [27, 28, 37]. Thus, catecholamines can contribute to pathological remodeling and alterations in cardiac contractile function. In addition, MPH has a mechanism of action similar to that of cocaine. It may induce an acute inflammatory response through its catecholamine-increasing effect, with an increase in lymphocytes and natural killer cells, which may have a direct cytotoxic effect on the heart [38].

Although the underlying mechanisms remain unclear, plausible biological explanations could account for the observed link between ADHD and cardiovascular diseases, such as immune system abnormalities, chronic inflammation, neuromodulator dysregulation, and dysregulation of the hypothalamic–pituitary–adrenal (HPA) axis [39, 40].

Of note, the risk of cardiovascular involvement in our patients warrants attention and highlights the importance of early detection tools such as STE, so careful follow-up evaluation is recommended during MPH treatment in pediatric patients with ADHD. Repeated studies on a larger sample size and for a prolonged period are also recommended to validate our results.

Limitations of the study: a small sample size and a short duration of follow-up are the main limitations of the study. Another potential limitation of the study is the lack of longitudinal follow-up after discontinuation of methylphenidate therapy. Therefore, it remains unclear whether the observed cardiac dysfunction represents transient, reversible changes or early indicators of persistent myocardial dysfunction. Assessing the reversibility of these findings would be of great clinical importance in determining the long-term cardiac safety of methylphenidate in pediatric patients.

## Conclusion

Subclinical cardiac systolic and diastolic alterations were detected in pediatric ADHD patients treated with methylphenidate. The safety of the drug on the heart is warranted; therefore, careful patient selection, baseline cardiovascular assessment, and follow-up of these patients are strongly recommended.

## Data Availability

all the data of the study are available from the corresponding author on reasonable request.

## Abbreviations

ADHD: Attention-deficit/hyperactivity disorder
BMI: Body mass index
BP: Blood pressure
DBP: Diastolic blood pressure
DSM-5: Diagnostic and Statistical Manual of Mental Disorders, Fifth Edition
EDV: End-diastolic volume
EF: Ejection fraction
ER: Emergency room
ESV: End-systolic volume
FS: Fraction shortening
GLS: Global longitudinal strain
HR: Heart rate
K-SADS: Kiddie Schedule for Affective Disorders and Schizophrenia
LV: Left ventricle
MPH: Methylphenidate
MPI: Myocardial performance index
RV: Right ventricle
SBP: Systolic blood pressure
SD: Standard deviation
TAPSE: Tricuspid annular plane systolic excursion
TDI: Tissue Doppler imaging
2D-STE: Two-dimensional speckle tracking echocardiography
